# Health and work-related factors as predictors of still being active in working life at age 66 and 72 in a Swedish population: A longitudinal study

**DOI:** 10.3233/WOR-220480

**Published:** 2023-12-15

**Authors:** Marie Bjuhr, Maria Engström, Anna-Karin Welmer, Sölve Elmståhl, Britt-Marie Sjölund

**Affiliations:** a Department of Caring Sciences, Faculty of Health and Occupational Studies, University of Gävle, Gävle, Sweden; b Department of Neurobiology, Care Sciences and Society (NVS), Aging Research Centre (ARC), Karolinska Institute, Stockholm, Sweden; c Stockholm University, Stockholm, Sweden; d Department of Clinical Sciences in Malmö, Division of Geriatric Medicine, Skåne University Hospital, Lund University, Malmö, Sweden

**Keywords:** Extended working life, healthy ageing, older people

## Abstract

**BACKGROUND:**

Health and work environment are known factors in being active in working life beyond legal retirement.

**OBJECTIVE:**

To investigate sociodemographic, health and work environment factors as possible predictors of being active in working life at ages 66 and 72. Secondly, investigate eventual changes over time, shortly after a major reform in the Swedish pension system, and predictors of still being active in working life at age 66.

**METHODS:**

We used a longitudinal design with two separate cohorts of people at age 60. One baseline assessment was made in 2001–2003 with two 6 years follow-ups, and one in 2007–2009 with one 6 years follow-up. Data were accessed through a Swedish national population-based study and analysed using logistic regression. To examine possible differences between the two cohorts, interaction terms with each independent variable were analysed.

**RESULTS:**

Being a man and working in a profession that requires at least three years of university education predicted that the person would still be active in working life at age 66 and 72. Additionally, having a light level of physical activity at work and being diagnosed with fewer than two diseases, also predicted still being active in working life at age 66. Only physical activity at work showed significant changes over time.

**CONCLUSION:**

Shortly after a major reform of the public pension system, there was an increase in participation in working life after age 66 and 72. However, gender, profession, and health factors are still important considerations regarding older people’s participation in working life.

## 1 Introduction

Due to the increased proportion of older people in the population, life expectancy and the number of healthier years [1], authorities have developed pension systems and other reforms to promote active, healthy ageing and extended working life [2–4]. Healthy ageing has been defined by the World Health Organization as ‘the process of developing and maintaining the functional ability that enables well-being in older age’ [5]. Factors important for successful and healthy ageing, from a lay perspective, include, e.g. being actively engaged in society, doing respected work and being able to work until the age of 67 [6].

Earlier research has described various factors related to a prolonged working life among older people. These factors can be categorized as: personal factors, e.g. gender, family circumstances, education and professional level; health-related factors, e.g. number of diagnosed diseases and self-reported health; and work-related factors, e.g. psychosocial aspects and physical activity at work [7–13].

According to the Organization for Economic Co-operation and Development (OECD), there is an overall increase in women participating in working life, but in most countries, older women still participate less in paid labour than older men [14]. With regard to family circumstances, research has shown that people who live alone are more likely to continue working beyond the eligible retirement age [15]. Moreover, extensive research has demonstrated that health status, which includes a self-rated health status [16–18] and/or objectively diagnosed disease(s) [16, 19], is an important factor in being active in working life at an older age. Furthermore, workers with a higher level of education are more likely to work beyond eligible retirement age [7–10], and they may have better opportunities to prolong working life compared to workers with a lower level of education [20, 21]. Working in professions that require a lower level of education may be associated with both poorer health and poorer working environments than working in a profession that requires a higher level of education [20]. Moreover, workers with a low income may not have a pension from their employment or have accumulated sufficient wealth to finance their own retirement [22]. Workers from higher paid professions may work longer because of better work conditions [22, 23]. Work environment aspects related to prolonged working life among older people often include work satisfaction [24] and job strain [11, 16–18], as well as physical load [16, 17]. An age-friendly work environment that promotes successful ageing may also have an impact on job satisfaction and a delayed retirement [12, 13, 25, 26].

It has been suggested that both health-related factors and job characteristics should be included in studies investigating older people’s participation in working life. In addition, testing should be done to see whether these factors may have changed over a period of time that encompasses changes in the pension system [24]. In Sweden, a new pension system aimed at retaining older people in working life was implemented successively between 1998 and 2003. This reform involved, e.g. increasing the age that people have the right to remain employed from 65 to 67 years. The result of this made it possible for a flexible age of retirement that was between 61 and 67 years during the period of this study. However, it was possible to work longer if there was an agreement to do so with the employer [2]. Despite extensive research investigating older peoples working life patterns that have often included attitudes and desired retirement planning; research has not included participants older than 65. Moreover, such research may not have included a longitudinal individual population-based sample or investigated changes in predictors associated with being active in working life after age 65 over a specified time period, i.e. shortly after a major reform of the public pension system.

The aims of the present study were first to investigate sociodemographic, health, and work environment factors as possible predictors of still being active in working life at ages 66 and 72. Secondly to investigate eventual changes over time in predictors of still being active in working life at age 66.

## 2 Methods

### 2.1 Data source and sample

The present study used a longitudinal design that included data from two of the four geographical regions and research centres that are a part of The Swedish National Study on Ageing and Care project (SNAC). SNAC is an ongoing population-based cohort study that was commissioned by the Swedish Ministry of Health and Social Affairs. When SNAC began in 2001, ethical approval was obtained from the Regional Research Ethics Committees at Karolinska Institute: KI reg.no 01-114 and reg.no 2013/828-31/3 and at Lund University: LU reg.no 128-00, reg.no 604-00 and reg.no 744-00. Individuals were randomly sampled from the National Population Registry and invited to participate. To obtain a representative sample from Sweden with demographic variation, SNAC is divided into four geographical regions that cover both urban and rural areas. In the SNAC project, there are 10 age cohorts and different age intervals using a stratified sampling procedure that also includes people living in institutions. A new cohort of participants aged 60 is included in SNAC every six years. In order to capture the transition from work to retirement, it was decided to include the age cohorts that began at 60 years. Using standardized protocols, professionals conducted the first data collection in 2000 through interviews and clinical examinations. A description of the national study has been published elsewhere [27]. The present study used data from two separate cohorts that were recruited six years apart. At recruitment, the participants were 60 years old. The inclusion criterion was that the participants were being active in working life to some extent. Persons who were unemployed or on full sick leave were excluded. Baseline data for Cohort 1 were collected during the period 2001–2003 (*n* = 479). There were two follow-ups for Cohort 1; one in 2007–2009 (*n* = 386), and the other in 2013–2015 (*n* = 320). For Cohort 2, baseline data were collected during the period 2007–2009 (*n* = 751), and in 2013–2015 (*n* = 615) for the one follow-up. Missing values and drop-outs due to a change of residence, not wanting to continue in the SNAC project, or death, resulted in fewer participants in the follow-ups.

### 2.2 Outcome variable

Being active in working life was assessed using 6- and 12- year follow-up data measured using the question: ‘what is your current occupational status’ with specifications as to whether the person was working and if so, the number of hours a week. Working at least one hour per week was considered as being active in working life. This is in accordance with one of the International Labour Organization’s (ILO) definitions of persons in employment [28].

### 2.3 Independent variable/predictors

The four personal factors were: gender, woman or man; living circumstances, living with someone or living alone; level of education, primary or lower secondary education, vocational/upper secondary education, or university; and professional level. According to the Swedish Standard of Occupations (SSYK), there are four qualification levels typical of a profession. Level 1 with no or low formal educational requirements, e.g. cleaning and home service staff. Level 2 with upper secondary education and post-secondary education shorter than two years, e.g. construction workers. Level 3 with practical or vocation-specific university or college education of at least 2–3 years, e.g. pharmacy dispensers. Level 4 with university education of at least three years, but typically four or more, e.g. civil engineers [29]. Due to the low proportion of respondents in SSYK Level 1, the variable was recoded into three options by collapsing Level 1 and Level 2 into one category.

Health related factors were measured using a self-reported health questionnaire with yes or no responses similar to Statistics Sweden’s (SCB) health index, and the number of diagnosed diseases. An overview of the included diagnosed diseases that were substantiated either by clinical examination performed by a physician or through an examination of the medical records is presented the supplementary file (Table 1). The total number of diseases was calculated and recoded into a dichotomous variable; none to one disease or two or more diseases. This was based on the consideration that in both cohorts, the median value of the number of diagnosed diseases was one disease.

**Table 1:** Description of participants’ activity in working life and number of hours per week

Cohort 1	60 years *N* = 479	66 years *N* = 479	72 years *N* = 386
Active in working life: Number (%)	All	*N* = 106 (27.5) *	*N* = 44 (13.8) **
	Number of hours/week^a^	Number of hours/week^b^	Number of hours/week^c^
Minimum– maximum	6–80	3–60	4–50
Mean	38.3	28.5	17.2
Percentiles:
25	37	17	8
50	40	30	12
75	40	40	25
Cohort 2	60 years *N* = 751	66 years *N* = 751
Active in working life: Number (%)	All	*N* = 204 (33.2) ***
	Number of hours/week ^d^	Number of hours/week ^e^
Minimum– maximum	4–80	1–80
Mean	37.6	27.9
Percentiles:
25	35	16
50	40	30
75	40	40

Note: Missing values (including dropouts) *N = 93, **N = 66, ***N = 136. Missing values: ^a^*N* = 27, ^b^*N* = 5, ^c^*N* = 1, ^d^*N* = 7, ^e^*N* = 9.

Work environment factors were measured with three yes or no questions: 1) Have you been exposed to something in your physical work environment/s that you believe can have affected your life or your health? 2) Has your workplace(s) been organized in a way that entailed great mental or physical strain? and 3) Have you had any negative experiences in your relations with superiors or work colleagues that you believe may have affected your life or health? Work satisfaction was measured with the question: How satisfying, do you consider, your work has been? That question had five response alternatives, and because there was a low proportion of the responses ‘very unsatisfying’, ‘unsatisfying’ or ‘neutral’, they were recoded into the dichotomous variables unsatisfying/neutral or satisfying. One question with three response alternatives ranging from light, medium or heavy measured the level of physical activity usually required during the person’s main occupation in their life.

### 2.4 Data analysis

The statistical analyses were performed using IBM SPSS Statistics 27. Descriptive statistics with frequencies and percentages were used to describe the characteristics, health status and work environment factors in the two cohorts. These were compared using cross-sectional analyses with Chi-Square statistics. For the longitudinal design, logistic regression analyses were used due to the binary outcome/dependent variable. Analyses examined: associations between being active in working life at age 66 and the independent variables with data from age 60, and at age 72 with data from age 60 regarding gender and level of education, and data from age 66 regarding living circumstances, health and work-related factors. First, all analyses were stratified by cohort. Initially, univariate unadjusted analyses were conducted. Then, analyses adjusted only for gender were performed. Thereafter analyses adjusted for gender and level of education, as well as analyses adjusted for gender and SSYK level were carried out. Secondly, the same analyses were performed on the whole sample, i.e. Cohorts 1 and 2 combined. To examine possible differences between the two cohorts, interaction terms, i.e. cohort multiplied with each independent variable (cohort*independent variable) were analysed. The results are presented as adjusted odds ratios (aOR) and 95% confidence intervals (CI) with the level of statistical significance set at *p* < 0.05.

The statistical analyses were performed using the Statistical Package for Social Sciences (SPSS) version 23.0 for WindowsResults. The baseline characteristics of the participants’ active in working life at age 60 are shown in Table 2. The proportions of participants with a higher educational level was significantly higher Cohort 2 than in Cohort 1 (*p*-value < 0.001). No significant differences between the two cohorts in gender, living situation, SSYK-level, health status and work environment factors were found. Analysis of participants’ health- status at baseline revealed, in both cohorts, higher proportions of having fewer diagnosed diseases and no self-reported ill-health. Regarding work environment factors, higher proportions reported having good work satisfaction vs. bad or neutral work satisfaction, also in both cohorts.

**Table 2:** Description of sociodemographic characteristics, health status and work environment factors and comparison between the two cohorts using chi-square statistics

	Cohort 1 *N* = 479	Cohort 2 *N* = 751	*p*-value
Characteristics: N* (%)
Gender woman	227 (47.4)	369 (49.1)	0.551
Living alone^a^	126 (26.4)	181 (24.1)	0.361
Level of education^b^			**<0.001**
Primary or lower secondary	109 (22.8)	159 (21.3)
Vocational/upper secondary	275 (57.4)	371 (49.6)
University	95 (19.8)	218 (29.1)
SSYK^c^ ^c^			0.051
Level 1 or 2	286 (59.7)	386 (52.6)
Level 3	71 (14.8)	127 (17.3)
Level 4	122 (25.5)	221 (30.1)
Health status: N (%)
None or one diagnosed disease	340 (71.0)	528 (70.3)	0.800
No self-reported ill health ^d^	350 (76.6)	528 (71.6)	0.060
Work environment: N (%)
Been exposed to something affecting your health in physical work environment^e^ No	321 (70.0)	523 (72.9)	0.359
Experiences at work that entailed mental or physical strain^f^ No	339 (73.4)	538 (73.4)	0.994
Negative experiences in relations with superiors/ work colleagues^g^ No	400 (86.2)	655 (89.4)	0.100
Level of physical activity required in work^h^			0.286
Heavy	119 (24.9)	159 (21.2)
Medium	114 (23.8)	180 (24.0)
Light	245 (51.3)	412 (54.9)
Self-reported good work satisfaction ^i^	423 (89.1)	661 (88.1)	0.623

Note: ^*^N = Number of participants. ^c^SSYK = Swedish Standard of Occupations. Missing values: ^a^Cohort 1 *N* = 2. ^b^Cohort 2 *N* = 3. ^c^Cohort 2 *N* = 17. ^d^Cohort 1 *N* = 22, Cohort 2 *N* = 14. ^e^Cohort 1 *N* = 23, Cohort 2 *N* = 18. ^f^Cohort 1 *N* = 17, Cohort 2 *N* = 18. ^g^Cohort 1 *N* = 15, Cohort 2 *N* = 18. ^h^Cohort 1 *N* = 1. ^i^ Cohort 1 *N* = 4, Cohort 2 *N* = 1.

## 3 Results

The mean value of reported number of working hours at age 66 was 28.5 hours in Cohort 1 and 27.9 hours in Cohort 2. The corresponding value at 72 years was 17.2 hours a week. See Table 1.

### 3.1 Description of personal health and work environment factors in each cohort

The baseline characteristics of the participants active in working life at age 60 are shown in Table 2. The proportions of participants with a university level of education were significantly higher in Cohort 2 than in Cohort 1 (*p*-value < 0.001). There were no significant differences found between the two cohorts regarding gender, living situation, SSYK level, health status or work environment factors. A descriptive analysis of the participants’ health status at baseline revealed that in both cohorts there were higher proportions of having none to one diagnosed disease vs. two or more diseases and of no self-reported ill health vs. self-reported ill health. With regard to work environment factors, there were higher proportions of reported good work satisfaction vs. bad or neutral work satisfaction, also in both cohorts.

### 3.2 Predictors of still being active in working life at age 66

The regression analyses showed that when working at age 60 in a profession with low educational requirements was compared to working in a profession at age 60 that requires at least three years, typically four or more years, of university education; the latter was a significant predictor of still being active in working life at age 66. The results were significant in both Cohort 1: aOR = 1.69, 95% CI = 1.01–2.82 and Cohort 2: aOR = 2.06, 95% CI = 1.41–3.02 (Table 3). When analysing the whole sample, i.e. Cohort 1 and 2 combined, the significant results remained (supplementary file, Table 5). Having university education, vs. primary or lower secondary education was a significant predictor in Cohort 2: aOR = 2.31, 95% CI = 1.39–2.83 (Table 3), as well as in the whole sample: aOR = 2.18, 95% CI = 1.45–3.27 (supplementary file, Table 5).

**Table 3:** Factors associated with being active in working life at age 66 using logistic regression analysis

	Cohort 1: N*=386 (active in working life *n* = 106)				Cohort 2: N**=615 (active in working life *n* = 204)			
Independent variables	aOR^∧^ (95% CI)	*p*-value	aOR^∧^^∧^ (95% CI)	*p*-value	aOR^∧^ (95% CI)	*p*-value	aOR^∧^^∧^ (95% CI)	*p*-value
Gender: Man (ref. woman)	1.20 (0.77–1.87)	0.431	1.23 (0.78–1.94)	0.366	1.36 (0.97–1.91)	0.072	1.37 (0.97–1.92)	0.073
Living with someone: (ref. living alone)	1.04 (0.63–1.73)	0.881	1.06 (0.64–1.77)	0.819	1.02 (0.68–1.52)	0.939	1.03 (0.68–1.55)	0.903
Education: (ref. primary or lower secondary)^a^
Vocational/upper secondary	1.25 (0.69–2.24)	0.462			1.46 (0.91–2.36)	0.119
University	1.81 (0.90–3.62)	0.095			2.31 (1.39–2.83)	**0.001**
SSYK^c^: (ref. Level 1 or 2) ^b^ Level 3	1.66 (0.87–3.16)	0.128			1.43 (0.89–2.27)	0.137
Level 4	1.69 (1.01–2.82)	**0.046**			2.06 (1.41–3.02)	**<0.001**
No more than one diagnosed disease: (ref. two or more diagnosed diseases)	1.63 (0.95–2.80)	0.077	1.65 (0.96–2.83)	0.073	1.57 (1.07–2.30)	**0.022**	1.48 (1.00–2.19)	0.051
Self-reported ill health: No (ref. yes)^c^	1.12 (0.65–1.92)	0.683	1.11 (0.64–1.92)	0.706	1.02 (0.70–1.49)	0.926	1.02 (0.70–1.50)	0.906
Exposed to something affecting your health in physical work environment: No (ref. yes) ^d^	1.30 (0.77–2.19)	0.33	1.34 (0.79–2.27)	0.278	1.11 (0.76–1.63)	0.589	1.11 (0.76–1.64)	0.585
Experiences at work that entailed mental or physical strain: No (ref. yes) ^e^	1.33 (0.79–2.26)	0.287	1.36 (0.80–2.31)	0.259	1.00 (0.68–1.46)	0.983	0.97 (0.66–1.43)	0.971
Negative experiences in relations with superiors/work colleagues: (ref. yes)^f^	1.25 (0.64–2.44)	0.516	1.31 (0.67–2.58)	0.432	1.01 (0.59–1.73)	0.979	1.10 (0.63–1.89)	0.755
Physical activity required in work: (ref. heavy)^g^
Medium	0.53 (0.25–1.10)	0.086	0.51 (0.24–1.06)	0.071	1.37 (0.81–2.33)	0.239	1.34 (0.78–2.29)	0.291
Light	1.43 (0.83–2.48)	0.201	1.30 (0.73–2.32)	0.372	1.59 (1.02–2.48)	**0.041**	1.40 (0.89 – 2.23)	0.149
Good work satisfaction: (ref. bad/neutral) ^h^	1.26 (0.58–2.77)	0.562	1.23 (0.56–2.72)	0.603	1.07 (0.63–1.84)	0.795	1.15 (0.67–1.98)	0.618

Note: * Number of participants, i.e. missing values from baseline (including dropouts) *N* = 93 and ** *N* = 136. ^∧^ Odds Ratio (95 % Confidence interval) adjusted for gender (gender unadjusted). ^∧^^∧^ Odds Ratio (95 % Confidence interval) adjusted for gender and level of education (gender only level of education). ^c^SSYK = Swedish Standard of Occupations. Missing values: ^a^Cohort 2, *N* = 3. ^b^Cohort 2, *N* = 1. ^c^Cohort 1, *N* = 14, Cohort 2, *N* = 14. ^d^Cohort 1, *N* = 17, Cohort 2, *N* = 16. ^e^Cohort 1, *N* = 11, Cohort 2, *N* = 15. ^f^Cohort 1, *N* = 10, Cohort 2, *N* = 15. ^g^Cohort 1, *N* = 1. ^h^Cohort 1, *N* = 4.

With regard to heath-status factors, regression analyses that were adjusted for gender, showed that having none to one diagnosed disease vs. two or more diagnosed diseases at age 60 was a significant predictor of still being active in working life at the age of 66 in Cohort 2: aOR = 1.57, 95% CI = 1.07–2.30. The result was not significant after adjusting for level of education (Table 3). When analysing the whole sample, the significant results remained after adjusting for both gender and level of education: aOR = 1.51, 95% CI = 1.10–2.07, as well as adjusting for gender and SSYK level: aOR = 1.51, 95% CI = 1.10–2.06 (supplementary file, Table 5).

After adjusting for gender, the work environment factor of having a light vs. heavy level of physical activity required in their main occupation at age 60, predicted still being active in working life at age 66 years in Cohort 2: aOR = 1.59, 95% CI = 1.02–2.48. However, the result was not significant after adjusting for level of education (Table 3). Similar results were found when analysing the whole sample (supplementary file, Table 5).

In the interaction analyses adjusted for gender, significant results for cohort*physical activity required in work were found: aOR = 2.55, 95% CI = 1.04–6.25. The odds of being active in working life at age 66 and having a medium level of physical activity required at work vs. heavy level, increased during the period studied. The significant results remained after adjusting for education and professional level. Non-significant results were found regarding all the other interaction terms, i.e. cohort*independent variable (supplementary file, Table 5).

### 3.3 Predictors of still being active in working life at age 72

The unadjusted regression analysis of being a man, vs. a woman significantly predicted still being active in working life at age 72: OR = 2.48, 95% CI 1.26–4.88. The result remained significant after adjusting for the level of education aOR = 2.58, 95% CI = 1.29–5.13 (Table 4). This was also true after adjusting for SSYK level aOR = 2.64, 95% CI = 1.32–5.28 (supplementary file, Table 3).

**Table 4:** Factors associated with being active in working life at age 72 using logistic regression analysis

Active in working life at age 72 N* = 320 (active in working life *N* = 44)
Independent variables	aOR^∧^ (95% CI)	*p*-value	aOR^∧^^∧^ (95% CI)	*p*-value
Gender: Man (ref. woman)	2.48 (1.26–4.88)	**0.009**	2.58 (1.29–5.13)	**0.007**
Living with someone: (ref. living alone) ^a^	0.80 (0.38–1.66)	0.541	0.79 (0.38–1.66)	0.531
Education: (ref. primary or lower secondary)
Vocational/upper secondary	1.14 (0.49–2.66)	0.757
University	2.28 (0.88–5.89)	0.089
SSYK^c^: (ref. Level 1 or 2) Level 3	1.48 (0.60–3.64)	0.396	1.47 (0.54–3.97)	0.543
Level 4	2.17 (1.05–4.51)	**0.038**	1.73 (0.56–5.31)	0.335
No more than one diagnosed disease: (ref. two or more diagnosed diseases)^b^	1.31 (0.66–2.60)	0.442	1.29 (0.65–2.58)	0.472
Self-reported ill health: no (ref. yes)^c^	1.32 (0.58–3.02)	0.512	1.22 (0.53–2.83)	0.638
Exposed to something affecting your health in physical work environment: no (ref. yes)^d^	1.35 (0.63–2.87)	0.438	1.32 (0.62–2.82)	0.47
Experiences at work that entailed mental or physical strain: no (ref. yes)^e^	1.09 (0.51–2.29)	0.83	1.08 (0.51–2.30)	0.834
Negative experiences in relations with superiors/work colleagues: no (ref. yes)^f^	0.96 (0.37–2.46)	0.93	1.05 (0.41–2.73)	0.919
Physical activity required in work: (ref. heavy)^g^:
Medium	0.80 (0.27–2.40)	0.689	0.74 (0.25–2.25)	0.579
Light	1.93 (0.84–4.41)	0.121	1.62 (0.67–3.92)	0.282
Work satisfaction: good (ref. bad/neutral)^h^	0.88 (0.31–2.46)	0.81	0.84 (0.30–2.39)	0.746

Note: *Number of participants, i.e. missing values (including dropouts) *N* = 66. ^∧^ Odds Ratio adjusted for gender (gender unadjusted). ^∧^^∧^ Odds Ratio adjusted for gender and level of education (gender only level of education), ^c^SSYK = Swedish Standard of Occupations. Missing cases: ^a^ *N* = 2, ^b^*N* = 9, ^c^*N* = 6, ^d^*N* = 16, ^e^*N* = 11.

When adjusting for gender, working in a profession that requires at least three years, typically four or more years, of university education significantly predicted still being active in working life at age 72: aOR 2.17, 95% CI = 1.05–4.51 (*p*-value 0.038) (Table 4). This is when compared to working in a profession with low education requirements.

## 4 Discussion

Overall, using the cohorts included in our study, our results showed that working in a profession that requires at least three years, typically four or more years, of university education significantly predicts that a person would still be active in working life at age 66. After adjusting for gender, this was also a significant predictor of still being active in working life at age 72. Having fewer diseases and a light vs. heavy level of physical activity at work predicated still being active in working life at age 66, although, this was only significant in Cohort 2. Being a man vs. a woman significantly increased the odds of being active in working life after age 65, i.e. at age 66 (cohort 1 and 2 combined) and at age 72.

We found that working in a high skilled profession increased the odds of still being active in working life at age 66 and at age 72. Furthermore, having a light level of physical activity at work also increased the odds of still being active in working life at age 66. These results are in line with findings from a Finnish study, which showed that employees in a higher vs. lower occupational class were twice as likely to continue working beyond eligible retirement age. Many of these differences were explained by having a physically light job in the highest occupational class [30]. Similar results were also found in a Dutch study that included employees aged 56–64 where a work environment factor such as lower physical workload predicted working beyond eligible retirement age [17].

We found that a clear majority of the participants had good health status at baseline. In 2016, the OECD reported that, among people aged 50–59 years across 14 European countries, the employment rate for those with two or more chronic diseases was 52% vs. 74% for those without a chronic disease [14]. Our study found that having fewer diagnosed diseases increased the odds of still being active in working life at age 66. These results are in line with previous research showing that better physical health and not having any chronic disease predicted prolonged working life [18, 19]. We did not find any significant results regarding associations between self-reported health and still being active in working life at age 66 or at age 72. However, a cross-sectional study among Swedish health-care workers aged 55–64 showed that poor self-related health was more strongly related to early retirement planning than was diagnosed diseases [31]. An interpretation of this could be that self-reported poor health is indicative of earlier retirement planning, but it does not necessarily predict working beyond age 65.

To investigate possible changes over time regarding predictors of still being active in working life at age 66, we included two different cohorts that were sampled six years apart. We found higher proportions of still being active in working life at age 66 in 2013–2015 than in 2007–2009. This could be interpreted to be a result of the changes in the pension reforms since the age at which a person has the right to remain employed was raised from 65 to 67 years. However, in the interaction analyses, cohort as an independent predictor of being active in working life was not significant. Physical activity required at work was the only significant interaction term revealed in our interaction analyses. Since there were no obvious changes over time, i.e. the six-year difference between our included cohorts, the timespan was possibly too modest.

It was only gender and professional level that significantly predicted being active in working life at age 72. This could suggest that at older ages, there are more complex circumstances surrounding being active in working life. Furthermore, in Sweden during the period studied, workers did not have the legal right to continue working after age 67; this was only possible with the employer’s consent [2], which may have reduced the importance of other factors.

Differences in existing working life that were found earlier between men and women have shown tendencies to become more equalized [32]. However, when investigating being active in working life at older ages, our results revealed that men had higher odds of being active in working life both at age 66 and 72 than women when they either were still fully employed or collected their old-age pension and at the same time continued to be employed for a minimum of one hour per week. This could be because women are more represented in human-to-human professions that include a great deal of emotional and physical strain. Additionally, women to a higher extent than men take responsibility for the care of close family members [33].

The OECD reported in 2016 on the development of greater opportunities regarding a healthier working life among the aging workforce [34]. Research has also shown that working life beyond pensionable age is often characterised as a period with retained favoured job characteristics that allow a flexible, rewarding, and less stress full working life [35, 36]. However, research has also pointed out the inequality in older workers’ abilities to extend their working life [37, 38]. More research looking into older people’s own experiences regarding their working life and how they interrelate with their health and well-being is needed. Our results reveal the difficulty of capturing these phenomena when only using a quantitative approach. For this reason, more research focusing on personal experiences is needed if we are to achieve a deeper understanding of this complex societal issue.

### 4.1 Limitations

The present study used data from SNAC, whose longitudinal design enabled temporal perspectives and strengthened our ability to infer causality. Nevertheless, our criterion only included participants who were active in working life at age 60, which led to the exclusion of people who were on full sick leave or unemployed. This may well explain the skewed distribution in the independent health status and work environment variables, which could be a limitation in this study. However, since the aim of the study was to investigate predictors of still being active in working life, we did not include participants who were not active in working life at age 60. Not being active in working life is defined as being fully retired, on full sick leave/disability pension, or unemployed. This grouping is similar to that made by Nilsson et al. (2016) [39]. The outcome measure, being active in working life, was defined as working at least one hour a week, which entailed a large discrepancy between the minimum and maximum values of those who were active in working life. However, it is important to emphasize that the majority, i.e. the 50 and 75 percentiles of those in both cohorts who were active in working life at the age of 66 worked 30–40 hours a week.

Furthermore, because some of the optional data were missing in two of the four SNAC sites, we accessed a smaller number of subjects, but the data still covered both rural and urban areas. Therefore, our study results may be generalized in Sweden, but maybe not on an international basis since the surrounding circumstances differ within different countries and cultures. However, the results may still be of international interest with regard to the heterogeneous conditions of those working beyond age 65. The proportion of dropouts with regard to the outcome, between the follow-up time points, varied between 17–19 %. The remaining, over 80 % of the study population, is considered to be an acceptable response rate [40]. Another limitation of this study is that we were not able to specify the proportions of the different reasons behind the dropouts.

When combining the two different cohorts with a six-year interval, and conducting analyses that include interaction terms, we found significant changes over time in only one of the 11 independent variables regarding predictors of still being active in working life at age 66. However, we could therefore combine those cohorts in the same analysis enabling more robust analyses with a larger sample when analysing factors predicting still being active in working life at age 66. Thus, the results from the analyses of Cohort 1 and Cohort 2 combined were the same as those for Cohort 2 alone, except for gender which became a significant predictor.

Another limitation of the present study is that we did not have access to sufficient data measuring financial incentives as a potential factor affecting people’s choice to work after age 65. The data in this study, as mentioned above, has been exported from a large register study, i.e. SNAC, which had not specifically envisioned the aim of investigating older people’s participation in working life at older ages. This may be considered a limitation since we only used available measurements and variables that were included in the standardized study protocol used in SNAC. However, the overall aim of the SNAC study is to create longitudinal databases, that cover both rural and urban areas in Sweden, broad aspects of aging among the population that is 60 years and older, and create the conditions for research and analysis of various issues that surround ageing after age 60. Using SNAC data made it possible to cover many aspects that are known to play a significant role regarding incentives behind still being active in working life after age 65, and substantial data regarding occupational status, working conditions, health, educational level, and present as well as past occupation were included.

## 5 Conclusion

Being a man, working in a high-skilled profession, having a light level of physical activity at work, and having fewer diseases predicted still being active in working life after age 65. This suggests that shortly after a major reform of the public pension system; gender, work, and health-related factors are still important considerations with regard to older people’s participation in working life. However, more research is needed to understand the interplay between health and being active in working life, especially at higher ages.
